# Effect of Orogastric Acetazolamide on Optic Nerve Sheath Diameter in Patients Undergoing Laparoscopic Donor Nephrectomies: A Randomized, Double-Blind Trial

**DOI:** 10.7759/cureus.24454

**Published:** 2022-04-25

**Authors:** Indu M Sen, Nitika Goel, Ashish Aditya, Ishwar Bhukkal, Ashish Sharma

**Affiliations:** 1 Anaesthesia and Intensive Care, Postgraduate Institute of Medical Education and Research, Chandigarh, IND; 2 Renal Transplant Surgery, Postgraduate Institute of Medical Education and Research, Chandigarh, IND

**Keywords:** intraocular pressure, laparoscopic donor nephrectomy, acetazolamide, intracranial pressure, optic nerve sheath diameter

## Abstract

Introduction

Laparoscopic Live Donor Nephrectomy(LLDN) is becoming an increasingly frequent procedure. The rise in intracranial pressure(ICP) during LLDN has not been measured yet. ICP can be evaluated by measuring ultrasonographic optic nerve sheath diameter(ONSD). Acetazolamide has been found to provide effective analgesia following LLDN. It also helps lowering the raised ICP. Therefore, we planned to study effect of orogastric Acetazolamide on ONSD in patients undergoing LLDN.

Methods

Forty Donors scheduled for LLDN were randomized preoperatively either into Group A receiving acetazolamide 5mg/kg or Group S receiving normal saline. ONSD was measured at time points:Time 0: In supine position before induction of GA, Time 1: 5 minutes after induction of GA but before giving orogastric acetazolamide, Time 2: 10 minutes after creating pneumoperitoneum, Time 3: 60 minutes after creating pneumoperitoneum, Time 4: Towards end of surgery, just before taking out specimen in modified flank position, Time 5: after extubating in supine position.

Results

Mean ONSD of left eye(4.42 ± 0.48) in Group S was significantly more than mean ONSD of left eye(4.16 ± 0.15; p-0.036) in Group A at 10 mins after creating pneumoperitoneum in modified flank position. Mean ONSD showed significant increase in group S at 10 and 60 minutes(4.374 ± 0.433mm in group S vs 4.151 ± 0.168 in group A; p-0.042 at 10 mins and 4.336 ± 0.301mm in group S vs 4.149 ± 0.282mm in group A; p-0.050 at 60 mins) after creating pneumoperitoneum as compared to group A.

Conclusion

Orogastric acetazolamide 5 mg/kg was found to be beneficial in preventing rise in ONSD from 10 minutes to 1 hour of creating pneumoperitoneum in patients undergoing laparoscopic donor nephrectomy under general anaesthesia. Acetazolamide was also found to be effective in reducing postoperative pain.

## Introduction

Laparoscopic live donor nephrectomy (LLDN) is the preferred surgical technique these days. Subjects undergoing LLDN are usually healthy individuals undergoing major surgery solely for the benefit of patients with end-stage renal disease (ESRD) [1]. Although laparoscopic nephrectomy is safer than open nephrectomy, the technique is not devoid of complications [1]. Pneumoperitoneum in laparoscopic nephrectomy has its own complications. There is a rise in intraabdominal pressure and in partial pressure of carbon dioxide (PaCO2) [2]. There can be a rise in intracranial/intraocular pressure and hemodynamic alterations [2]. Among the multimodal pain management techniques available for LLDN patients, single-dose orogastric acetazolamide has been found to be effective in reducing referred pain postoperatively [3]. Acetazolamide is a reversible inhibitor of the carbonic anhydrase enzyme and has been found to lower intraocular pressure (IOP) by decreasing aqueous humor production [4]. Currently, ultrasonography (USG) is being used as a non-invasive technique to measure optic nerve sheath diameter (ONSD), which reflects an increase in IOP thereby ICP [5]. Literature on the effect of CO2 pneumoperitoneum on intraocular pressure as measured by ONSD is limited. Furthermore, the effect of single-dose orogastric acetazolamide on ONSD is also not known Therefore, this present study was planned to assess and compare ONSD in patients receiving orogastric acetazolamide versus those receiving placebo.

## Materials and methods

This prospective, randomized, double-blind study was conducted in the Department of Anesthesia and Intensive Care and the Department of Renal Transplant Surgery of the Postgraduate Institute of Medical Education and Research, Chandigarh. After institutional ethics committee approval (NK/2316/MD/112339-40, dated 29.12.2016), 40 American Society of Anesthesiologists (ASA) I/II live renal donors, aged between 18 and 60 years, scheduled to undergo laparoscopic LDN under general anesthesia (GA) were enrolled in the study. Written informed consent was taken from all the participants. The exclusion criteria included the presence of any ocular defects/wounds or previous ocular surgery, any previous history of hypertension, patients having double renal arteries, laparoscopic nephrectomies converted to open surgeries, and refusal to consent.

Preoperatively, all the donors were evaluated to assess their fitness for the proposed surgical procedure under general anesthesia. Donors were kept NPO for eight hours for solid food, six hours for unclear fluids, and two hours for clear fluids. They were premedicated with tab. ranitidine 150 mg and tab. alprazolam 0.25 mg two hours prior to surgery. Randomization of patients was done using coded sealed envelopes, which were computer-generated, where five blocks of eight each were formed, and subsequently, participants were allocated to one of the two groups of 20 patients each. Both the participants and investigators were blinded to study medications till the completion of statistical analysis. All the investigators involved in patient management and data collection, including the patient’s anesthesiologist administering general anesthesia, were unaware of the group assignment. Group A donors received orogastric acetazolamide 5 mg/kg in 10 ml NS followed by 10 ml NS for flush soon after the induction of anesthesia through an orogastric tube while group S donors received 10 ml NS through an orogastric tube followed by 10 ml NS for flush soon after the induction of anesthesia through the orogastric tube.

In the operating room, patients were monitored for heart rate (HR), blood pressure (BP), continuous electrocardiogram (ECG), arterial oxygen saturation (SpO2), end-tidal carbon dioxide (EtCO2), and temperature using multichannel monitors (Datex-Ohmeda S/5 Avance, General Electric, Schenectady, NY). General anesthesia was administered to all the participants using a standard anesthetic technique. After pre-oxygenation with 100% oxygen, anesthesia was induced with inj. morphine 0.1 mgkg-1 and propofol 2-3 mgkg-1 (titrated to effect). Vecuronium 0.1 mgkg-1 was given to facilitate tracheal intubation.

Following the induction of general anesthesia and the administration of intravenous cefuroxime 1.5 gm antibiotic prophylaxis, a Foley’s catheter was placed in the bladder. An oral-gastric tube was inserted and kept in place until the completion of the procedure. Patients were placed in the modified flank position with the torso in the left lateral position and secured to the table. The arms were flexed and placed at chest level with appropriate axillary and lower extremity padding. Pneumoperitoneum was created using a Veress needle and the peritoneal cavity is insufflated to maintain an intraabdominal pressure (IAP) between 12 and 15 mmHg.

Acetazolamide 5 mgkg-1 diluted in 10 ml normal saline, administered through an orogastric tube soon after the induction of anesthesia, followed by flushing with 10 ml of normal saline to 50% of subjects in a computer-generated randomized fashion. Anesthesia was maintained with isoflurane in O2/N2O 30:70 targeting a minimum alveolar concentration of 1-1.3. End-tidal carbon dioxide was maintained within 32-36 mmHg throughout the surgery. The patients received a bolus of vecuronium 1 mg at regular intervals as guided by the train of four (TOF) response. Heart rate and mean arterial pressure were maintained within 20% of the baseline values. Intraoperatively, adequate hydration was maintained by intravenous infusion of 10-15 ml/kg/hour of balanced solution along with titrated doses of mannitol (0.5 g/kg). All patients received a bilateral ultrasound-guided transverse abdominis plane (TAP) block with bupivacaine 0.5%; 15 ml on each side. Thereafter, the residual neuromuscular blockade was reversed with inj. neostigmine 50 µgkg-1 and inj. glycopyrrolate 10 µgkg-1. On return to consciousness and adequate recovery of muscle strength, patients were extubated and shifted to the post-anesthetic care unit (PACU).

ONSD measurement

Patients were placed supine for baseline ONSD reading and then in the modified flank position for further readings. After applying gel over the closed upper eyelid, a high-frequency (5-10 MHz) ultrasound probe (Sonosite, Inc. Bothell. WA) was placed on the temporal area of the eyelid, the hand holding the probe stabilized on the forehead of the patient, to prevent excessive pressure over the eye.

The placement of the probe was adjusted to give a suitable angle for displaying the entry of the optic nerve into the globe. ONSD was measured 3 mm behind the globe using the electronic ultrasonographic caliper and an axis perpendicular to the optic nerve. For each optic nerve, three measurements were taken in a transverse plane. A total of six readings were taken for each eye at the following time points:

Time 0: In supine position before induction of GA

Time 1: Five minutes after induction of GA but before giving orogastric acetazolamide

Time 2: Ten minutes after creating pneumoperitoneum in the modified flank position

Time 3: Sixty minutes after creating pneumoperitoneum in the modified flank position.

Time 4: Toward the end of the surgery, just before taking out the specimen in the modified flank position

Time 5: After extubating in the supine position

In the postoperative room, patients were monitored for mean arterial pressure (MAP) and heart rate (HR). Postoperative pain was assessed using the comfort score [6] and visual analog scale (VAS) [7]. Patients having a comfort score of ≤ 6 at an instant were assessed for incisional site pain and referred pain (at Rest, Movement, and Cough). We used the comfort score to avoid any negative feedback to the patient by using the positive words “are you comfortable” instead of negative words like “are you in pain”. This decreases the incidence of false reporting of pain. For assessment of postoperative pain, VAS was used where 0 stands for no pain and 10 stands for worst imaginable pain [7]. Episodes of nausea/vomiting were recorded in the postoperative care unit by the prevention of postoperative nausea and vomiting (PONV) score [8] at regular intervals (30 mins, 60 mins, 4 hr, 12 hr, 24 hr, 48 hr, and 72 hr) by an investigator blinded to group allocation. The intensity of parietal and visceral pain at rest (supine), on movement (sitting up from supine), on coughing, and shoulder pain was noted when the patient’s comfort score was ≤6. Rescue analgesia (intravenous injection tramadol 1.5 mg/kg) was given when the comfort score was ≤6. Patients having persistent pain even after 30 minutes of intravenous tramadol administration were given intravenous inj. morphine sulphate 0.1 mg/kg (second rescue analgesic agent). A total dose of inj. morphine was converted into tramadol equivalents. We also recorded the total number of patients requiring rescue analgesia. Patients complaining of severe nausea or occurrence of an episode of vomiting received rescue antiemetics in the form of intravenous metoclopramide 10 mg. Persisted nausea or vomiting after giving inj. metoclopramide was treated with 4 mg of inj. ondansetron as the second rescue antiemetic. Side effects like shivering, pruritus, nausea/vomiting, dizziness, and respiratory depression were noted during the study period.

Study outcomes

The primary outcome was to compare ONSD values in two groups undergoing LLDN under GA. The secondary outcome was to compare the changes in hemodynamic parameters and the need for rescue analgesia in the first 72 hours.

Statistical analysis

Descriptive as well as inferential statistical analysis of the study subjects was done by using Statistical Package for the Social Sciences (SPSS, version 22.0; IBM Corp., Armonk, NY)/Microsoft Excel 2010 software (Microsoft Corporation, Redmond, WA). For continuous variables, mean and SD was described and for categorical variables, proportions were calculated amongst groups. The normalcy of these variables was checked using the Kolmogorov Smirnov test. The independent T-test/Mann-Whitney test (whichever is applicable) was applied to compare the above-mentioned parameters between the two groups. Repeated measure (RM) analysis of variance (ANOVA) was applied to compare these parameters according to the time period, e.g. 0 min, 15 min, 30 min, 45 min, 60 min, etc., between two study groups. If any RM ANOVA results came statistically significant, the posthoc test (Tukey test) was applied to find out particular time events. In addition, the association of categorical variables, e.g. gender and PONV, was calculated by applying the chi-square (Fisher’s exact, if applicable) test. Standard binary logistic regression was applied to find out any independent association between the study groups and ONSD after controlling factors that would have a p-value of ≤0.10 in univariate analysis. A two-tailed p-value of ≤0.05 will be considered statistically significant with a 95% confidence interval.

Considering a 50% less increment in ONSD value in the study group compared to the placebo group with a power of 80% and a confidence interval of 95%, 16 patients were required in each group [9]. However, considering the possibility of dropouts, we enrolled 20 patients in each group.

## Results

**Figure 1:**
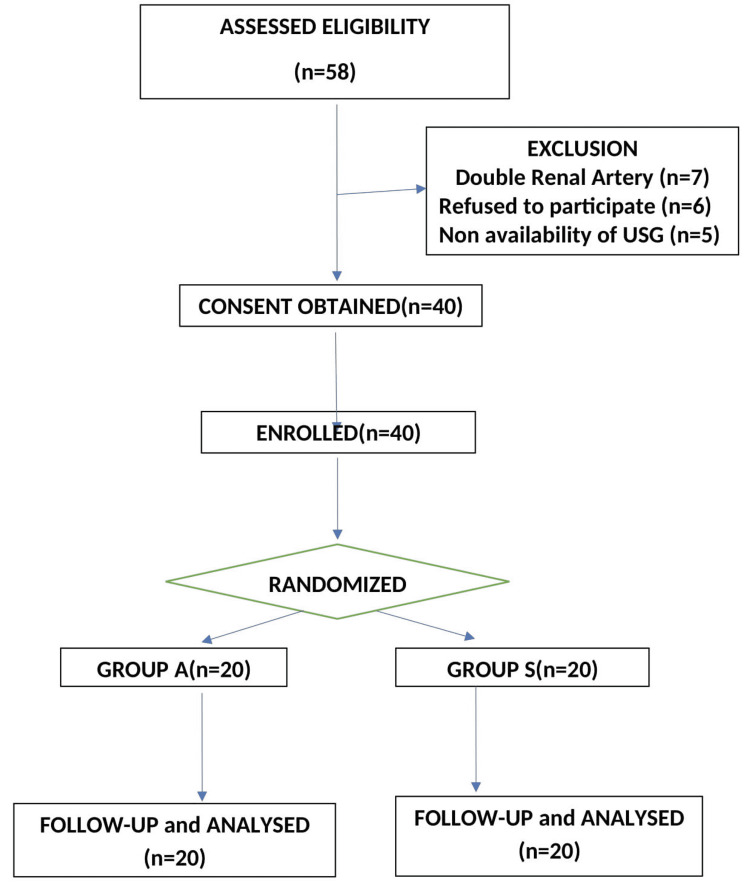
CONSORT flow diagram for the selection of patients Patient flowchart

A total of 247 subjects underwent LLDN during the study period, out of which 58 donors were assessed for eligibility. Eighteen donors could not be enrolled, as they didn’t match the inclusion criteria (seven patients were found to have double renal artery, six patients refused to participate, and five cases could not be enrolled because of the non-availability of an ultrasound machine). Forty patients were finally recruited for the study. The study flow diagram is shown in Figure 1. Demographic parameters, duration of surgery, and anesthesia were comparable in both groups (Table 1).

**Table 1:** Demographic parameters and intraoperative variables Values are expressed in mean±SD (in mm) or absolute numbers; p<0.05 is significant SD: standard deviation

PARAMETERS	GROUP A(N=20)	GROUP S(N=20)	P VALUE
Age(years)	45.80 ± 10.175	42.30 ± 9.815	0.275
Sex (female)	17	15	0.695
Height (cms)	165.10 ± 4.141	164.75 ± 8.058	0.864
Weight (kgs)	60.60 ± 8.53	62.15 ± 9.69	0.595
BMI	22.21±3.04	22.80±2.24	0.502
Duration of Surgery (Min)	201.25 ± 16.38	200.75 ± 20.22	0.932
Duration of Anaesthesia (Min)	239.75 ± 18.74	242.65 ± 21.10	0.648

Primary outcome

**Figure 2:**
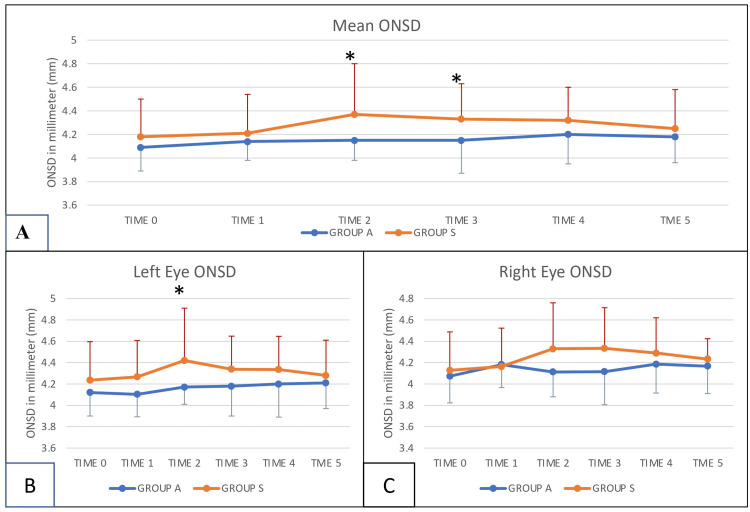
A: Comparison of the mean ONSD between Group A and Group S; B: Intergroup comparison of left eye ONSD between Group A and Group S; C: Intergroup comparison of right eye ONSD between Group A and Group S Measurement was done at the following points: Time 0: In the supine position before induction of general anesthesia (GA) Time 1: 5 minutes after the induction of GA but before giving orogastric acetazolamide Time 2: 10 minutes after creating pneumoperitoneum in the modified flank position Time 3: 60 minutes after creating pneumoperitoneum in the modified flank position Time 4: Toward the end of the surgery, just before taking out the specimen in the modified flank position Time 5: After extubation in the supine position *p<0.05; ONSD: optic nerve sheath diameter

ONSD of the right and left eyes were assessed and compared within groups and between both groups. There was no significant difference (P > 0.05) in the mean value of ONSD between the right and left eyes in any of the groups at any time point. Intergroup comparison of ONSD of the right and left eyes was also done. The mean ONSD of the left eye (4.42 ± 0.48) in Group S was significantly more than the mean ONSD of the left eye (4.16 ± 0.15) in Group A at Time 2, i.e., 10 minutes after creating pneumoperitoneum (Figure 2B). There was no difference in the mean ONSD of the right eyes between both groups (Figure 2C). During the study, it was observed that taking measurements of ONSD in the dependent eye, i.e., the right eye, is a bit difficult because of the position and could reflect minimal variation in readings. Thus, the mean ONSD of the right and left eyes was also taken for comparison between Group A and Group S at all time points. The baseline mean ONSD in Group A was 4.09 ± 0.19 (mm) and in Group S, it was 4.18 ± 0.32 (mm), which was comparable in both groups (p=0.312). There was a significant increase in ONSD in Group S at Time 2 and Time 3, i.e., 10 and 60 minutes after creating pneumoperitoneum (p-0.042 and 0.05 at 10 and 60 minutes, respectively) (Figure 2A, Table 2, Table 3).

**Table 2:** ONSD in Groups A and S p-value ≤ 0.05 is significant; values are expressed in mean±SD (in mm); SD: standard deviation; ONSD: optic nerve sheath diameter

TIME	ONSD IN GROUP A		P-VALUE	ONSD IN GROUP S		P-VALUE	MEAN ONSD		P-VALUE
	RIGHT EYE	LEFT EYE		RIGHT EYE	LEFT EYE		GROUP A	GROUP S	
TIME 0	4.073 ± 0.250	4.12 ± 0.220	0.440	4.128 ± 0.352	4.236 ± 0.335	0.071	4.096 ± 0.195	4.182 ± 0.319	0.312
TIME 1	4.181 ± 0.214	4.103 ± 0.207	0.195	4.163 ± 0.362	4.267 ± 0.340	0.072	4.142 ± 0.165	4.215 ± 0.329	0.379
TIME 2	4.113 ± 0.233	4.170 ± 0.156	0.447	4.329 ± 0.434	4.420 ± 0.488	0.221	4.151 ± 0.168	4.374 ± 0.433	0.042
TIME 3	4.116 ± 0.310	4.183 ± 0.285	0.134	4.334 ± 0.374	4.338 ± 0.307	0.963	4.149 ± 0.282	4.336 ± 0.301	0.050
TIME 4	4.186 ± 0.271	4.201 ± 0.314	0.832	4.290 ± 0.328	4.336 ± 0.309	0.252	4.193 ± 0.248	4.326 ± 0.285	0.126
TIME 5	4.168 ± 0.257	4.206 ± 0.236	0.454	4.234 ± 0.188	4.271 ± 0.326	0.564	4.187 ± 0.220	4.252 ± 0.225	0.358

**Table 3:** Right and left eye ONSD between Group A and Group S p-value ≤ 0.05 is significant; values are expressed in mean±SD (in mm); SD: standard deviation; ONSD: optic nerve sheath diameter

TIME	RIGHT EYE ONSD		P-VALUE	LEFT EYE ONSD		P-VALUE
	GROUP A	GROUP S		GROUP A	GROUP S	
TIME 0	4.073 ± 0.250	4.128 ± 0.352	0.573	4.120 ± 0.220	4.236 ± 0.335	0.201
TIME 1	4.181 ± 0.214	4.163 ± 0.362	0.848	4.103 ± 0.207	4.267 ± 0.340	0.072
TIME 2	4.133 ± 0.233	4.330 ± 0.434	0.063	4.169 ± 0.156	4.420 ± 0.488	0.036
TIME 3	4.116 ± 0.310	4.334 ± 0.374	0.052	4.183 ± 0.285	4.338 ± 0.307	0.106
TIME 4	4.186 ± 0.271	4.290 ± 0.328	0.285	4.201 ± 0.314	4.336 ± 0.309	0.103
TIME 5	4.168 ± 0.257	4.234 ± 0.188	0.360	4.206 ± 0.236	4.271 ± 0.326	0.474

Secondary outcome

**Figure 3:**
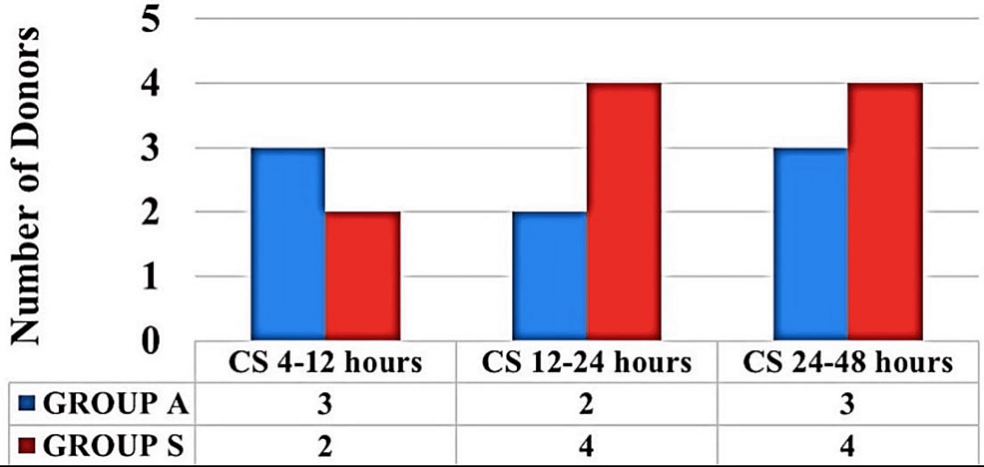
Number of donors with a comfort score less than or equal to 6 CS: comfort score

There was no significant difference in comfort score between both the groups at any time frame postoperatively. During the time intervals of 0-30 mins, 30-60 mins, 60 mins-4 hours, 48-72 hours, all the donors in both the groups had a comfort score of >6, thus VAS was not assessed. Between four and 12 hours, Group A had a comfort score of 5 (5-7), versus Group S with a comfort score of 4 (4-6) (p 0.228). Between 12-24 hours, Group A had a comfort score of 6 (6-7) versus Group S with a comfort score of 5 (4-7) (p 0.260), and between 24 and 48 hours, Group A had a comfort score of 6 (6-7) versus Group S with a comfort score of 5 (5-7) (p 0.087). A number of donors who had a comfort score of ≤6 were assessed for pain using VAS (Figure 3).

VAS for parietal and visceral abdominal pain during movement and coughing was significantly less in Group A at 12-24 hours postoperatively (Tables 4-5). Shoulder pain was present in a total of eight donors out of 40 after surgery. Five donors in Group S and three donors in Group A reported shoulder pain. There was no significant difference in the number of donors complaining of shoulder pain (p>0.05). Median VAS was significantly less in group A at 12-24 hours and 24-48 hours (Group A median VAS 2.5 (1.30-2.70) versus Group B median VAS 5.20 (4.85-5.35) (p=0.024)). At 24-48 hours, Group A had a median VAS of 1.70 (1.50-2.10) while Group S had a VAS of 4.20 (3.95-4.75) (p=0.034).

**Table 4:** Comparison of visual analog scale for parietal pain on rest, movement, and coughing PP: parietal pain; VAS: visual analog score; R: rest; M: movement; C: cough Data expressed as median (IQR – interquartile range); p<0.05 is significant

Time (Hours)	GROUP A (n=20)	GROUP S (n=20)	P-VALUE
VAS R (4 – 12)	2.75 (2.62-3.48)	2.30 (1.8 – 3.8)	0.480
VAS M (4 – 12)	3.6 (3.5 – 4.0 )	3.90 (3.0 – 4.1)	0.827
VAS C (4 – 12)	4.2 (3.8 – 4.2 )	3.70 (3.10 – 4.5)	0.507
VAS R (12 – 24)	3.2 ( 1.9 – 3.5)	2.70 (2.05 – 2.75)	0.453
VAS M (12 – 24)	3.8 (2.5 - 3.9 )	4.50 (4.35 – 5.20)	0.024
VAS C (12 – 24)	3.5 (3.4 – 3.7 )	4.70 (4.45 – 5.1)	0.025
VAS R (24-48)	2.5 (2.05 – 2.97)	2.15 (1.57 – 2.95)	0.381
VAS M (24-48)	3.7 (3.4 – 4.25)	3.05 (2.82 – 3.27)	0.221
VAS C (24-48)	3.9 (3.90 – 4.45)	3.15 (3.02 – 4.17)	0.146

**Table 5:** Comparison of VP on visual analog score for abdomen on rest, movement, and cough VP: visceral pain; VAS: visual analog score; R: rest; M: movement; C: cough Data expressed as median (IQR – interquartile range); p<0.05 is significant

Time(Hours)	GROUP A (n=20)	GROUP S (n=20)	P VALUE
VAS R (4 – 12)	3.75 (3.60-3.9)	2.30 (1.8 – 4.3)	0.471
VAS M (4 – 12)	4.3 (3.9 - 4.4 )	3.90 (2.8 – 4.8)	0.658
VAS C (4 – 12)	4.6 (4.2 – 4.8)	4.40 (3.4 – 4.5)	0.275
VAS R (12 – 24)	4.1 ( 2.3 – 4.4)	2.60 (2.45 – 2.95)	0.456
VAS M (12 – 24)	2.6 (2.1 – 2.8)	4.60 (3.9 – 5.20)	0.025
VAS C (12 – 24)	3.1 (2.9 – 3.5)	4.60 (3.9 – 4.9)	0.025
VAS R (24-48)	3.25 (2.42 – 4.0)	2.15 (2.1 – 3.17)	0.081
VAS M (24-48)	3.8 (3.12 – 5.52)	3.25 (3.12 – 3.75)	0.559
VAS C (24-48)	4.45 (3.95 – 5.07)	3.65 (3.42 – 4.17)	0.059

The total dose of tramadol as rescue analgesia, frequency of doses, and time for first and second rescue analgesia were comparable in both groups (p>0.05) (Table 6 ). The incidence of nausea and vomiting was comparable between the two groups (Group A: 20%, Group S: 15%, p 0.24). Rescue antiemetics were given to two patients in Group A and one patient in Group S. Regarding the hemodynamic parameters, the mean heart rate and mean blood pressure were comparable between the two groups throughout all ONSD measurements.

**Table 6:** Comparison of postoperative rescue analgesia Data expressed as median (IQR – interquartile range) or absolute numbers

VARIABLE	Group A (n=20)	Group S (n=20)	P Value
Amount of rescue analgesia (mg)	140 (93-182)	115 (83 – 210)	0.948
No. of patients requiring rescue analgesia	6	8	0.447
Time for 1^st^ rescue analgesia (hours)	12 (4-18)	12 (4-24)	0.698
Time for 2^nd^ rescue analgesia (hours)	13 (5-21)	14 (6-24)	0.665

## Discussion

In this prospective, randomized, double-blind study, the effect of single-dose orogastric acetazolamide 5 mg/kg on ONSD was assessed. Laparoscopic live donor nephrectomies are routinely being performed nowadays for retrieval of kidneys from healthy donors [10]. Despite the clear benefits of laparoscopic live donor nephrectomy over conventional open nephrectomy like a short hospital stay, small incision, etc., subjects are exposed to the usual risk of laparoscopies like bleeding and the delayed complications of hypertension, paraesthesia, incisional hernia, and small bowel obstruction [10]. Raised ICP is a well-known complication of acute rise in intraabdominal pressure as in laparoscopy [11]. The possible proposed mechanisms include decreased lumbar venous plexus blood flow (leading to increased cerebrospinal fluid (CSF) pressure), increased PaCO2 (resulting in increased cerebral blood flow), and decreased cerebral venous outflow.

The CSF in the dural sheath of the optic nerve is connected to the CSF in the intracranial subarachnoid space. The changes in the intracranial pressure are directly conducted to the fluid in the optic nerve sheath because of the incompressible nature of CSF. The arachnoid surrounding the optic nerve approx. 3 mm proximal to the fovea has a highly ramified meshwork of delicate trabeculae [12]. The optic nerve sheath is most distensible at this point due to elastic subarachnoidal trabecular anatomy, thus making it the best point for ONSD interpretation. Killer et al. evaluated the efficacy of sonographic ONSD in estimating ICP. They showed ONSD as a strong and accurate predicting factor for increased ICP, with sensitivity and specificity of 100% [13]. In another study by Sahu et al., a significant correlation of ONSD with ICP (r = 0.532, p = 0.002) was found. An ONSD threshold of 5.5 mm predicted ICP > 20 mmHg with high sensitivity (100%) and specificity (75%) (area under receiver operating characteristic (ROC) curve = 0.904, p=0.01) in their study [14].

Acetazolamide, being a carbonic anhydrase inhibitor, has been found to reduce pain by reducing the carbonic acid formation responsible for causing pain during laparoscopy [15]. Similarly, acetazolamide is known to decrease CSF flow rate and CSF production by inhibiting the carbonic anhydrase enzyme in the choroid plexus [16], thus reducing intracranial pressure. As previously mentioned, ONSD is a known surrogate marker of Intracranial pressure. The effect of laparoscopy and acetazolamide on intracranial pressure will be reflected as changes in ONSD.

Verdonck et al. studied the changes in optic nerve sheath diameter in patients undergoing robotic-assisted laparoscopic radical prostatectomy in a steep head-down position and observed no change in optic nerve sheath diameter, although ICP and CVP increased significantly but in the compensatory limits in individuals not having intracranial pathology [17]. Kim et al. also measured ONSD in two different groups of patients kept in the Trendelenburg and reverse Trendelenburg position and observed that ONSD increases slightly up to 15 minutes and found no significant change in ONSD because of position [18]. A study conducted in robotic-assisted laparoscopic radical prostatectomy concluded that long-time head down during laparoscopy increases IOP [19]. A systemic review of evidence from different surgical specialties like the spinal, neurosurgical, and urological fields explored the changes in intraocular pressure according to patient positioning [20]. The study reported a significant rise in IOP in the head-down position. It was also observed that IOP rises in a time-dependent fashion in all the studies.

In the present study, all patients were kept in the modified flank position. The right eye, being dependent on left laparoscopic live donor nephrectomy, was expected to have a significant change in intraocular pressure. Changes in ONSD were also measured as expecting the increased intracranial pressure due to pneumoperitoneum. Ten minutes after creating pneumoperitoneum, significantly high mean ONSD values were observed in the left eye Group S versus left eye Group A. High ONSD values in Group S were also observed 60 mins after the creation of pneumoperitoneum. The baseline mean optic nerve sheath diameter was comparable between the left and right eyes in both groups. Hemodynamic parameters were stable and comparable between both groups.

In the present study, the comfort score was noted and VAS for parietal and visceral pain was assessed only when patients were not comfortable. We chose to use the comfort score because using negative words like ‘pain,’ during assessment as compared to more positive words like ‘comfort’ increases the incidence of false reported pain. No significant difference in comfort score in both groups at any time frame was noted in the immediate postoperative period. This is because all patients were given ultrasound-guided transversus abdominis plane block. At times 0-30 minutes, 30-60 minutes, 60 minutes to 4 hours, and 48-72 hours, donors had a satisfactory comfort score of more than 6. VAS assessment of abdominal pain (visceral and parietal) and shoulder pain for patients having comfort score ≤ 6 at 4-12 hours, 12-24 hours, and 24-48 hours postoperatively revealed significantly less VAS score in Group A for both visceral and parietal pain on movement and coughing in the 12-24 hour period. There was a significant difference in VAS shoulder tip pain at the time intervals of 12-24 hours and 24-48 hours postoperatively. The incidence of postoperative nausea and vomiting was similar in both groups.

Limitations

Though ultrasound measurement of ONSD has become an established non-invasive method of measuring changes in the ICP, it was not compared with the gold standard, which includes the placement of invasive ICP probes. However, the placement of invasive ICP probes in donor patients was not reasonable in our study. Therefore, we feel more research on non-invasive modalities for ICP measurement is still required, especially in the perioperative settings. Furthermore, there was no direct measurement of IAP in our study. Though the carbon dioxide insufflating pressure was kept between 12 and 15mmHg, it is not a true indicator of intraabdominal pressure. Direct measurement of IAP using intravesical catheters was not performed in our study. Though we obtained a rise in ONSD after the creation of pneumoperitoneum, we could not find a definitive correlation between IAP and ONSD. Thus, we feel future studies incorporating the direct IAP measurement method should be done for a better correlation between ONSD and IAP. Third, all donors in our study received a bilateral TAP block. Therefore, we feel that the VAS scores may be confounded by the analgesic effect of the TAP block.

## Conclusions

Thus, we conclude orogastric acetazolamide 5 mg/kg was found to be beneficial in preventing a significant increase in optic nerve sheath diameter from 10 minutes to one hour of creating pneumoperitoneum in patients undergoing laparoscopic donor nephrectomy under general anesthesia. Acetazolamide was also found to be effective in reducing postoperative pain in donor patients, especially during movement and coughing. Further trials can be conducted using acetazolamide as preventive therapy for reducing intraocular and intracranial pressure in other laparoscopic surgeries expected to have a longer duration of surgery with a steep head-down position.
